# *Dirofilaria repens* transmission in southeastern Finland

**DOI:** 10.1186/s13071-017-2499-4

**Published:** 2017-11-10

**Authors:** Risto Pietikäinen, Stig Nordling, Sakari Jokiranta, Seppo Saari, Petra Heikkinen, Chris Gardiner, Anne-Marie Kerttula, Tiina Kantanen, Anna Nikanorova, Sauli Laaksonen, Antti Lavikainen, Antti Oksanen

**Affiliations:** 1 Kymenlaakso Central Hospital, Kotkantie 41, 48210 Kotka, Finland; 2 Faculty of Medicine, Department of Pathology, University of Helsinki, P.O. Box 21, 00014 University of Helsinki, Helsinki, Finland; 3 Department of Microbiology and Immunology, University of Helsinki, P.O. Box 21, 00014 University of Helsinki, Helsinki, Finland; 4 Faculty of Veterinary Medicine, Department of Veterinary Biosciences, University of Helsinki, P.O. Box 66, 00014 University of Helsinki, Helsinki, Finland; 5 Finnish Food Safety Authority Evira, Wildlife and aquatic pathology (FINPAR), Elektroniikkatie 3, 90590 Oulu, Finland; 6 Veterinary Pathology Services, Joint Pathology Center, 606 Stephen Sitter Ave. Silver Spring, Silver Spring, MD 20910-1290 USA; 7 HUSLAB, Division of Clinical Microbiology, P.O. Box 720, 00029 HUS, Helsinki, Finland; 8 HUSLAB, Division of Pathology, P.O. Box 720, 00029 HUS, Helsinki, Finland; 9 The Moscow State Academy of Veterinary Medicine and Biotechnology named after KI Skryabin, Akademika Skryabina Street, 23 Moscow, Russia

**Keywords:** Vector-borne nematodes, Autochthonous infection, Climate change, Zoonosis, Dirofilariosis

## Abstract

**Background:**

The spread of vector-borne diseases to new regions has become a global threat due to climate change, increasing traffic, and movement of people and animals. *Dirofilaria repens*, the canine subcutaneous filarioid nematode, has expanded its distribution range northward during the last decades. The northernmost European locations, where the parasite life-cycle has been confirmed, are Estonia and the Novgorod Region in Russia.

**Results:**

Herein, we describe an autochthonous *D. repens* infection in a Finnish woman. We also present two cases of *D. repens* infection in imported dogs indicating the life-cycle in the Russian Vyborg and St Petersburg areas, close to the Finnish border.

**Conclusions:**

The most obvious limiting factor of the northern distribution of *D. repens* is the summer temperature, due to the temperature-dependent development of larvae in vectors. With continuing climate change, further spread of *D. repens* in Fennoscandia can be expected.

## Background

Species of *Dirofilaria* (Spirurida, Onchocercidae) are vector-borne filarioid nematode parasites. Worldwide, filarioid nematodoses with major public health importance include lymphatic filariosis and onchocercosis, while dirofilariosis, setariosis and onchocercosis cause major animal health hazards and have economic implications [1]. Animal parasitic filarioids are often also zoonotic. There is recent evidence documenting the expansion of some animal diseases associated with filarioid parasites to boreal and sub-Arctic areas, including Finland [2, 3], and at northern latitudes, species of several filarioid genera are now known to be emerging [4, 5].

*Dirofilaria immitis*, the dog heartworm, and *Dirofilaria repens*, the canine subcutaneous worm, use mosquitoes as vectors. The host specificity of these parasites is not high and they are found occasionally in foxes and cats as well as sporadically in other mammal species, including humans [6, 7]. Since potential mosquito vectors are available virtually over all the sub-Arctic, and many arctic regions, the northern border of distribution of these *Dirofilaria* species is not determined by the availability of the vectors but rather by the ability of microfilariae to mature into infectious larvae in the mosquito vector [8–10], as maturation is temperature-dependent. *Dirofilaria repens* development in mosquitoes to the infective stage takes 8–13 days at 28–30 °C, 10–11 days at 26 °C and 16–20 days at 22 °C [11]. At temperatures below 14 °C, *D. immitis* larval development ceases [12], but restarts if the temperature rises. When a mosquito with infective stage (L3) larvae feeds on a human being, it may infect this aberrant host [13]. Both subcutaneous, periorbital and eyelid human infections caused by *D. repens* occur in endemic areas [14].

**Fig. 1:**
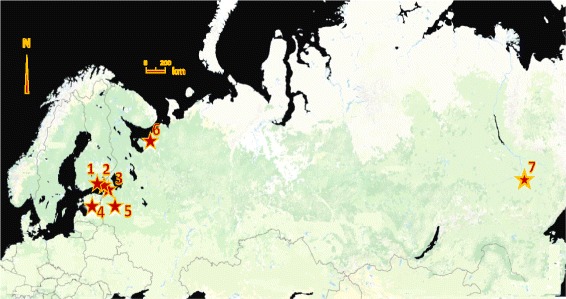
The northernmost *Dirofilaria repens* infections found reported. See text for further information of the reports. 1, Hamina (61°N, 27°E); 2, Vyborg (61°N, 20°E); 3, St Petersburg (60°N, 30°E); 4, Tartu (58°N, 27°E); 5, Novgorod Oblast (58°N, 33°E); 6, Arkhangelsk (65°N, 41°E); 7, Yakutsk (62°N, 130°E)

*Dirofilaria repens* is endemic in dogs in Africa, southeastern Asia, and the Mediterranean region [15]. In Europe, its distribution range has recently expanded northward, assumedly partly due to climate change [8, 16–18]. In a 2012 publication, the European Scientific Counsel Companion Animal Parasites [19] reported the parasite to have established in Austria, Germany and Poland. It has also been found in Lithuania in dogs [20], and in Latvia, in both dogs and humans [21, 22]. Thus far, the northernmost European site where the parasite life-cycle has been confirmed to take place is Estonia, where *D. repens* microfilaraemia was seen in three dogs in 2013–2014 [23]. In European Russia, the reported northern limit of *D. repens* is in the Novgorod Region [24]. According to Russian veterinary practitioners, however, dirofilariosis occurs in the dog population of Saint Petersburg, as was also reported on a veterinary clinic website (Anna Nikanorova, pers. comm. 2017). The Novgorod Region and the southern part of the Leningrad Region are located approximately at the same latitude as Estonia (Fig. 1). Recently, three cases of human ocular dirofilariosis were reported from Saint Petersburg [25, 26]. In one case, the patient had reportedly not left the region during the previous three years, suggesting endemicity of *D. repens* in the Leningrad Region. In addition, a single case of dirofilariosis was reported in 1999 in the city of Arkhangelsk [27]. A 15 year-old child was suspected to have been infected in Arkhangelsk, but because of travel history to Sochi and Rostov-on-Don, the origin of infection could not be confirmed. In Yakutsk, Siberia, located over permafrost and known as the coldest city on earth, three *D. repens* infections were reported in dogs and one in a cat [28]. Moreover, six cases of *D. immitis* infection were diagnosed in dogs and one in a fox between 1996 and 2010 [28].

In Finland, autochthonous *D. repens* cases in dogs have not been diagnosed previously, but a dog imported from Romania was found infected in 2014 [29]. Another species, *Dirofilaria ursi*, a parasite of the brown bear, using black flies as vectors and having lower temperature requirements for development in its vector [30], is prevalent in Finnish bears at least in Northern Karelia (unpublished pers. obs.).

Human dirofilariosis has been increasingly reported in the past few years and thus should be considered an emerging zoonosis in many parts of the world [13, 31, 32]. Here, we report in a human being the first autochthonous *D. repens* infection in Finland. The infected patient lived in Hamina, southeastern Finland (Fig. 1), which is around 35 km from the Russian border. We document the surgical removal of the parasite nodule and the identification of the adult, gravid female nematode and its microfilariae. Further, we discuss possible transmission pathways. Moreover, we report two cases in dogs imported to Finland from St Petersburg and Vyborg, Russia, the latter just 25 km from the Finnish border and 90 km from Hamina.

### Autochthonous human case, southeastern Finland

In March 2015, a 70-year-old Finnish woman, with no recent travel history abroad, sought medical care due to erythema, swelling and some itching on the volar side of her left forearm. Suspecting an allergic reaction, the general practitioner prescribed a single dose of oral corticosteroid and a course of antihistamine. After four days, antibacterial cephalexin chemotherapy was initiated because the symptoms had not abated and painful nodules were observed in the forearm. The absolute leucocyte count (13.1 × 10^9^/l; norm 3.4–8.2 × 10^9^/l), eosinophil leukocyte count (1.2 × 10^9^/l; norm 0.03–0.1 × 10^9^/l) and C-reactive protein concentration (22 mg/l; norm <3 mg/l) were elevated. Soon after starting cephalexin, the patient got a possible allergic reaction, with flush over the face and upper chest, and the antibiotic was replaced with piperacillin and tazobactam for three days and, after that, continued with clindamycin. Over the next two weeks, the pain and swelling disappeared, but a thumb tip size nodule remained in the forearm. In early April, 20 days after the first visit to the general practitioner, the nodule was surgically removed for histological examination. The excision wound healed normally leaving only a minor scar.

**Fig. 2:**
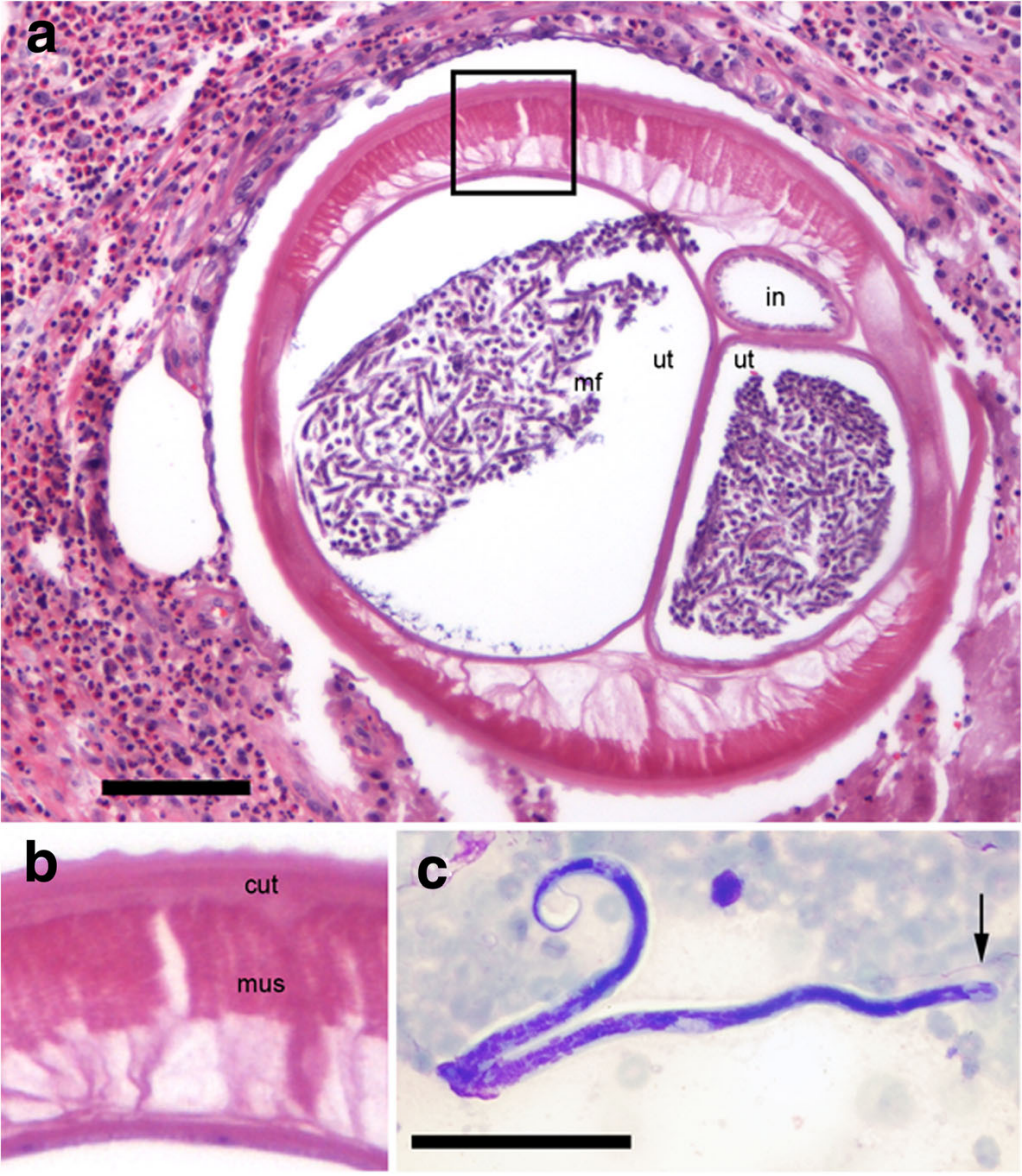
Histopathology as seen in the subcutaneous lesion (**a** and **b**) and microfilaria found in the blood smear (**c**) from the human patient, a 70 year-old female. A diffuse dermal inflammatory infiltrate composed of eosinophilic granulocytes and histiocytes surrounding an adult female filarioid nematode can be observed in (**a**). The area in the rectangle is enlarged in (**b**). A cuticle with evenly spaced external longitudinal cuticular ridges, a musculature of coelomyarian type, a small intestine and a paired uterus filled with microfilariae were considered typical of *Dirofilaria* spp. Haematoxylin-eosin stained histological section. A microfilaria possessing three nuclei in a large cephalic space (*arrow*) can be observed in (**c**). *Abbreviations*: cut, cuticle; mus, musculature; in, intestine; ut, uterus; mf, microfilariae. *Scale-bars*: **a**, 100 μm; **b**, 50 μm

Upon histological study, an inflammatory reaction composed of eosinophilic granulocytes and histiocytes surrounding a nematode was noted. Morphology revealed at least one gravid, adult female filarioid nematode, having a cuticle with evenly spaced external longitudinal cuticular ridges. The musculature was coelomyarian (muscle fibers in a cylindrical pattern) and polymyarian (many cells in each quadrant of a cross-section), and the intestine was very small (Fig. 2). The nematode had two large uteri filled with microfilariae. These findings were characteristic of *Dirofilaria* spp. The external longitudinal cuticular ridges ruled out *D. immitis*, but we could not distinguish between other potential species. Since *D. repens* is present in Estonia, and the brown bear parasite *D. ursi* is prevalent in eastern Finland, we aimed at specific diagnosis by both morphological examination of microfilariae and molecular identification.

Three weeks after the parasitic nodule had been excised, diurnal and nocturnal whole heparine blood samples were collected for microscopic analysis of possible microfilariae, concentrated using a standard filtration method with Millipore filters [33], and stained on microscope slides with Giemsa. In the nocturnal samples, microfilariae were detected with a maximum of 2 mf/ml. Two months after the excision, 1 mf/8 ml was still detected, and in August, 4.5 months after the excision, no microfilariae were found from the patient.

The microfilariae possessed 2 or 3 nuclei in the cephalic space (Fig. 2). The length of 10 microfilariae was measured, which was not easy since caudal filaments of crooked microfilariae were not clearly visible (Fig. 2). Length measurements, 200–260 μm, agreed with that reported for *D. ursi* in bears (198–242 μm) [34]. Microfilariae of *D. repens* in dogs have been reported to be longer (323 ± 21 μm) [35], or, using a somewhat different Knott’s method, even longer (369 ± 11 μm) [36].

### Infection in two dogs imported from Vyborg and St Petersburg

We also report here two confirmed cases in dogs imported to Finland from Russia, one from Vyborg and another from St Petersburg, located about 25 km and 160 km from the Finnish border, respectively.

In May 2013, a 2 year-old Rabbit Dachshund, imported from St Petersburg, Russia, more than a year earlier, was brought to a veterinary clinic in Turku with vomiting and presenting signs of abdominal pain. Laboratory examinations revealed, apparently unrelated to the presented illness, many mf in a Giemsa-stained thin blood film. Microfilariae were about 370 μm in length, possessed rather short cephalic space terminating by a pair of nuclei separate from the remaining somatic nuclei of the microfilaria. These morphological features were considered typical of *D. repens*.

In 2015, a 15 month-old mixed breed bitch imported from Vyborg was presented for a routine ovariohysterectomy in Helsinki. During the surgery, the veterinary surgeon found a wormlike organism about 2 cm long squirming in the abdominal cavity. The surgeon preserved the worm in alcohol and sent it for identification. Based on anamnestic information, *D. repens* was suspected, but as the sample represented an immature nematode lacking diagnostic morphological features, the specimen was submitted for molecular identification.

### Molecular identification

For a specific diagnosis, DNA was isolated from a blood sample containing mf from the human patient and from the ethanol-preserved worm specimen from the second canine case. DNA of three adult *D. ursi* specimens, from three different brown bears from eastern Finland, was used as a reference. Partial mitochondrial sequences of the cytochrome *c* oxidase subunit 1 (*cox*1) gene and *12S* ribosomal DNA (rDNA) were amplified using previously reported filarioid-specific primers [37, 38]. Standard Sanger sequencing of the amplicons confirmed the causative agent to be *D. repens* in both the human and canine cases. The sequences (*cox*1, 649 bp; *12S* rDNA, 468 bp) of the human patient were identical with several published sequences of *D. repens* in GenBank (e.g. *cox*1, Estonian canine isolate, accession no. KR780980; *12S* rDNA, Russian human isolate, KM205372). The *cox*1 sequence of the canine specimen and that of the human patient were identical, and the identity in *12S* rDNA was 99%. In both sequence regions of the human and canine specimens, the identity to *D. ursi* was 92%. The sequence data are available in the GenBank database under the accession numbers KY828978–KY828986.

## Discussion

The patient was a resident of the town of Hamina, on the southern coast of Finland and about 35 km from the Russian border, living in a suburban environment. She said she had stayed within Finnish territory since her last visit to Tallinn, Estonia, four years prior. As *D. repens* has been reported to cause symptoms to humans within one year of infection [13], it is probable that she got infected in the summer of 2014, which she spent mostly in Hamina and at a location approximately 50 km from Hamina (35 km from Russian border). She practiced outdoor activities, such as collecting forest berries and mushrooms and hiking in the forest. Mosquitoes were abundant in these environments.

We identified the nematode from the patient’s subcutaneous tissue and its mf in the circulating blood morphologically as a species of *Dirofilaria*, and the molecular methods unequivocally showed the worm to be *D. repens*. If we had based the diagnosis on mf morphology alone, possibly we would have misidentified those as microfilariae of *D. ursi*. The dwarfism of microfilariae, ~20–40% shorter than those described from dogs [35], might indicate weakness and non-viability; the patient might have been a dead end host for the parasite with no transmission of infection possible further from her. Genchi et al. [11] state that, usually, *D. repens* parasites do not mature to adults in humans, but knew of three microfilaremic humans in Europe and one in Iran. Later, an Indian case was also reported [39].

The mean summer temperature at Kotka meteorological station (25 km from Hamina) in summer 2014 was 16.8 °C, which is 0.7 °C warmer than the mean summer temperature in 1980–2010 (http://ilmatieteenlaitos.fi/tilastoja-vuodesta-1961). Warm summers are known to promote the emergence of outbreaks of filarioid nematodes in arctic ungulates [4, 40]. It appears that the conditions in summer 2014 may have been more favourable than average, enabling the development of *D. repens* to L3 larvae in mosquito vectors. The origin of *D. repens* in a mosquito in Hamina region is unknown, but two alternative hypothetical options are present. First, the nematode life-cycle might have established in the region perhaps years earlier. However, Finnish Food Safety Authority Evira annually collects hundreds of red foxes and raccoon dogs from southeastern Finland for other disease surveillance (especially rabies antibody and *Echinococcus multilocularis*), and has never identified *D. repens* in them (unpublished pers. obs.). Yet, the surveillance is not specifically aimed at *D. repens*, and its prevalence in foxes and raccoon dogs, somewhat aberrant hosts even in an endemic area, would not be expected to be high [6].

The other possibility is that the patient was infected by a mosquito which had carried the infection to Hamina from abroad. For example, it is possible that strong air currents might have blown mosquitoes from the Baltic States over the Gulf of Finland in summer 2014. *Aedes punctor* mosquitoes have been reported to be able to fly at least 46 km in still air and some evidence exists that on long flights mosquitoes take advantage of wind [41]. However, most mosquito species appear to have an average flight range of between just 25 m and 6 km [42].

The final transport of infected mosquitoes could also have been by cargo ship to the Port of Hamina, a major ship terminal in Finland. Passive transportation by vehicles is regarded to be the most important mode of *Aedes albopictus* long-distance dispersal within Europe [43].

The finding of *D. repens* in dogs imported from Vyborg and St Petersburg indicates that the parasite has gained a foothold in close proximity of the Finnish border in the Karelian Isthmus. Thus, spreading from there and settling in southeastern Finland would be the simplest explanation. In Finland, heartworm preventive treatment of dogs is not common practice.

Although *D. immitis* is still regarded as a southern species in Europe, mostly restricted to the Mediterranean region [19], in Yakutsk, both *D. repens* and *D. immitis* thrive in spite of extremely cold winters. During the winter, the worms have homoeothermic conditions within the canine host. During the summers, when transmission potentially occurs, Yakutsk is warm (1961–1990 mean in July 18.7 °C, https://www.yr.no/place/Russia/Sakha/Yakutsk/statistics.html). Therefore, the 130 *Dirofilaria* Development Units (DDUs) (degree-days above 14 °C) proposed to be required for development into the infective stage in mosquitoes [8, 11] can probably be reached within the mosquito lifespan during the summer; thus the low winter temperature is irrelevant.

## Conclusions

It is evident that the patient reported here became infected by *D. repens* within the Finnish territory. Consideration of all the vector dispersal mechanisms, combined with climate change assessments, suggests that further expansion of *D. repens* in Finland, and perhaps other northern countries, is possible. In addition, it is probable that almost any filarioid nematodes parasitizing animals can, under appropriate circumstances, infect humans and undergo some degree of development [44, 45]. This topic is also highly timely, since the predicted change in temperature indicates a particularly strong warming trend at the high latitudes in the northern hemisphere, which may increase the incidence of climate-sensitive arthropod-borne diseases such as filarioses (see [4, 35, 46, 47]). The parasites should be taken into account in human and animal disease diagnostics and in vector monitoring.

## Abbreviations

*cox*1: Cytochrome *c* oxidase subunit 1
DDU: *Dirofilaria* development unit, degree days exceeding 14 °C
Mf: Microfilaria
